# Interpreting sulci on hominin endocasts: old hypotheses and new findings

**DOI:** 10.3389/fnhum.2014.00134

**Published:** 2014-05-01

**Authors:** Dean Falk

**Affiliations:** ^1^Department of Anthropology, Florida State UniversityTallahassee, FL, USA; ^2^School for Advanced ResearchSanta Fe, NM, USA

**Keywords:** Affenspalte, endocasts, lunate sulcus, MH1, middle frontal sulcus, prefrontal cortex, Stw 505, Taung

## Abstract

Paleoneurologists analyze internal casts (endocasts) of fossilized braincases, which provide information about the size, shape and, to a limited degree, sulcal patterns reproduced from impressions left by the surface of the brain. When interpreted in light of comparative data from the brains of living apes and humans, sulcal patterns reproduced on hominin endocasts provide important information for studying the evolution of the cerebral cortex and cognition in human ancestors. Here, new evidence is discussed for the evolution of sulcal patterns associated with cortical reorganization in three parts of the hominin brain: (1) the parietotemporo-occipital association cortex, (2) Broca's speech area, and (3) dorsolateral prefrontal association cortex. Of the three regions, the evidence regarding the last is the clearest. Compared to great apes, *Australopithecus* endocasts reproduce a clear middle frontal sulcus in the dorsolateral prefrontal cortex that is derived toward the human condition. This finding is consistent with data from comparative cytoarchitectural studies of ape and human brains as well as shape analyses of australopithecine endocasts. The comparative and direct evidence for all three regions suggests that hominin brain reorganization was underway by at least the time of *Australopithecus africanus* (~2.5 to 3.0 mya), despite the ape-sized brains of these hominins, and that it entailed expansion of both rostral and caudal association cortices.

## Introduction

Paleoneurologists study fossilized skulls and internal casts of their braincases (endocasts) in order to glean information about the evolution of the size, shape, and surface morphology of brains. Although endocasts may yield information about the sulci that delimit the gyri and larger convolutions of the cerebral cortex, the degree to which sulcal patterns are reproduced on primate (including hominin) endocasts varies with species (smaller-brained species produce clearer endocasts than larger-brained closely related species), age of the individual (infants and mature individuals produce less detailed endocasts than individuals of other ages), geological conditions (e.g., “natural endocasts” that occur in limeworks sites in South Africa are relatively detailed compared to artificially or electronically prepared ones), and luck. Sulcal patterns are not generally well reproduced on hominin endocasts, perhaps partly because the meninges prevent the brain from leaving detailed impressions on the inner walls of the braincase. However, the smaller endocasts of australopithecines produce more detail than endocasts from larger-brained hominins. Identification of the few sulci that are reproduced on hominin endocasts is subject to interpretation, which is facilitated by comparison with sulcal patterns from brains of apes and humans.

Because paleoneurologists attempt to interpret functionally the bumps and occasional sulci that may be reproduced unclearly or only partially on the surfaces of endocasts, they are sometimes described as engaging in the debunked pseudoscience of phrenology. Although endocasts are the only *direct* evidence that can shed light on the evolution of the hominin cerebral cortex, the anecdotal association of paleoneurology with phrenology does little to encourage neuroscientists to study them. This is unfortunate because studies of hominin endocasts sometimes indicate fruitful directions for new comparative cytoarchitectonic studies that could, potentially, advance our understanding of hominin brain evolution. For example, neuroscientists are currently investigating whether or not ape and human brains exhibit asymmetries in the cytoarchitecture and volumes of Brodmann's areas (BA) 44 and 45, which comprise Broca's speech area in the left hemisphere of humans (Schenker et al., 2010), partly because of gross anatomical shape asymmetries that appear on endocasts (Holloway et al., 2004a, pp. 31–32). Along similar lines, new research on frontal lobe morphology on endocasts from *Australopithecus*, presented below, suggests that it would be informative for neuroscientists to conduct comparative cytoarchitectonic studies on BA 47, which constitutes the orbital operculum of humans.

Paleoneurology has also been influenced by an unfortunate tradition of “paleopolitics,” which continues to hamper progress in the field (Falk, 2011). As just one example, the implications of the hypothesis that widespread association cortices evolved in concert (Dart, 1929; Finlay and Darlington, 1995; de Winter and Oxnard, 2001; Falk, 2007, 2009), rather than in a piecemeal “mosaic” fashion (Barton and Harvey, 2000; Holloway, 2001), have yet to be considered in the debate about whether or not the brains of early hominins were initially reorganized solely in the posterior parts of their brains. As detailed in section The Affenspalte (“ape sulcus”), this particular debate and the paleopolitics that continue to drive it (Falk, 2011) were seeded over a century ago when Grafton Elliot Smith changed the name of a sulcus that is found in ape brains.

Below, the limited but direct evidence from sulcal patterns is evaluated with respect to the evolution of association cortices in three different parts of the hominin brain, beginning with the back of the cerebral cortex and moving forward. Section The Affenspalte (“ape sulcus”) discusses a very old hypothesis about the caudal shift of the visual cortex on the lateral surface of the brain during hominin evolution and questions, in light of new evidence, whether neurological reorganization of posterior association cortices can be evaluated on hominin endocasts from a so-called lunate sulcus that borders the rostral boundary of primary visual cortex in extant apes. Moving forward in the brain, section Broca's Area reviews new findings from research on the comparative cytoarchitecture of the inferior frontal gyrus in apes and humans, including Broca's area in the latter, and the implications of this research for studies of hominin endocasts. Finally, section Prefrontal Cortex provides new research on endocasts from *Australopithecus*, including *Australopithecus sediba*, which suggests that the middle frontal sulcus of the frontal lobe and BA 47 would be fruitful foci for future comparative cytoarchitectonic research.

## The affenspalte (“ape sulcus”)

**Figure 1:**
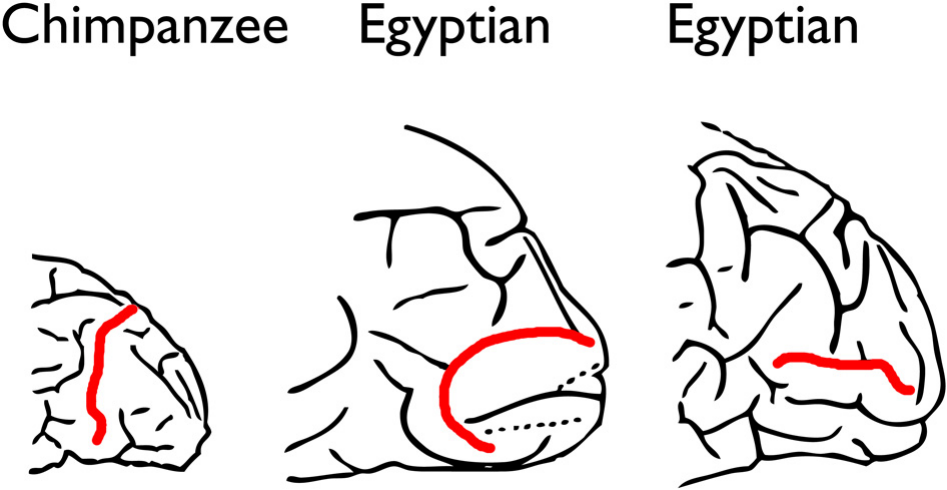
**The Affenspalte of a typical chimpanzee brain compared with sulci in human brains that Grafton Elliot Smith identified as homologs of the Affenspalte.** Because Smith thought the name Affenspalte (“ape sulcus”) was inappropriate for humans, he changed it to “lunate sulcus” to be more inclusive. The illustrations represent posterior ends of left hemispheres, and the Affenspalte and so-called lunate sulci are in red. The middle and right figures are modified from Smith (1903, pp. 75 and 81).

One hundred and eleven years ago, Grafton Elliot Smith compared the gross sulcal patterns on the lateral surfaces of the occipital lobes of over 400 Egyptian and “Soudanese Negro” human hemispheres to an almost equal number of combined monkey and ape (“simian”) hemispheres (Smith, 1903, 1904a). Smith claimed that the human brains had sulci that were homologous with the large crescent-shaped sulcus called the Affenspalte that closely approximates the rostral boundary of primary visual cortex (BA 17, V1) in apes, Old World monkeys, and some New World monkeys (Figure 1). In particular, he emphasized that the sulcal patterns in his human sample closely resembled those of gorillas, but also noted that the “resemblances to the Simian pattern… is not quite so obvious… in European types of brain,” which explained “the common belief in the absence of the supposedly distinctively Simian sulci on the lateral aspect of the occipital region of the human brain” (Smith, 1904a, p. 437). Smith believed the term Affenspalte (ape sulcus) was a misnomer because the sulcus was present in humans as well as simians. He, thus, renamed it the “sulcus occipitalis lunatus” or, more simply, the lunate sulcus (Smith, 1903, p. 76).

However, Smith also observed that lunate sulci of humans, especially those with a “European type of brain” (Smith, 1904b, p. 63), were more caudal than those of apes, a migration he attributed to evolutionary expansion of association cortex located rostral to BA 17. Although Affenspalte of monkeys and apes are typically long and arced, the features that Smith identified as lunate sulci on human brains were not only further back in the brain, but also extremely variable (Figure 1):

“The lunate sulcus may extend right across the lateral aspect of the hemisphere from the dorso-mesidal to the ventro-lateral edge, as in most Chimpanzees. It may be a much shorter furrow placed anywhere between these two extremes. It may be transverse, oblique or horizontal in direction. It is very frequently interrupted by a submerged “gyrus translunatus”: (which) occasionally … comes to the surface and completely divides the lunate sulcus into a pars dorsalis and a pars ventralis. Either of these fragments may be joined to a sulcus praelunatus so as to form a pattern, which is at first sight… perplexing” (Smith, 1904a, p. 448).

Despite the fact that the sulci he identified as lunate sulci in humans were neither typically crescent-shaped nor oriented in the direction typical for the Affenspalte, Smith concluded, “We can state with absolute certainy (sic) that the so-called “Affenspalte” is not the exclusive property of the Apes because it certainly is present in the great majority of, if not in all, human brains” (Smith, 1903, p. 83).

In 1904, Smith bolstered his controversial argument that the Affenspalte of apes was homologous with his newly identified lunate sulcus in humans by asserting that both closely approximated the rostrolateral borders of primary visual cortex. Smith based his claim on the stripe of Gennari, which consists of white fibers that run underneath and parallel to the surface of the cerebral cortex in BA 17. This white stripe is visible to the naked eye in sectioned brains: “I have examined the distribution of the stria Gennari in relation to the furrow which, from a mere study of the surface, seemed to be the sulcus lunatus in more than 200 human brains and a large series of (monkeys and) Apes and have been able to demonstrate its value as the one decisive criterion in the sure identification of the “Affenspalte” (Smith, 1904a, pp. 440–441). This statement was illustrated with a schematic of a horizontal section through the right cerebral hemisphere of an Egyptian brain.

Over 2 years later, however, Smith suggested his observations were from coronal sections taken about 1 centimeter behind the parieto-occipital fossa of nearly 200 human hemispheres, rather than from horizontal sections (Smith, 1907). (Perhaps Smith sectioned right hemispheres of humans horizontally and their corresponding left hemispheres coronally, but this is not clear.) Surprisingly, Smith claimed he could map at least eight different cortical areas by visual inspection alone of fresh brains: “In the process of mapping out any given area one can deal with a large piece of brain, and by making incisions with a scalpel at right angles to its borders can trace its edge exactly, however irregular its outline may be, while still retaining intact the actual tissue of the region to be mapped; whereas by other methods … the area (must) be cut up into sections… By means of the macroscopic examination of fresh material, it is possible to obtain results… from at least 200 specimens in the same time that it takes to examine one by the histological method” (Smith, 1907, pp. 198–199). Clearly, the methods Smith used to examine the relationship of the stripe of Gennari to the so-called lunate sulcus in humans were, at best, ambiguous.

Much to his credit, however, Smith correctly hypothesized that the primary visual cortex shifted caudally along the lateral surface of human occipital lobes in conjunction with evolutionary expansion of adjacent parietotemporo-occipital association cortices. Further, the implication that ape brains with more rostral Affenspaltes have relatively greater volumes of BA 17 than apes with more caudal ones has recently been confirmed (de Sousa et al., 2010). Smith's suggestion that sulci are homologous in related species if they delimit the same cortical areas (in this case, BA 17) was also valid. On the other hand, a contemporary high-resolution MRI study of 220 human hemispheres shows conclusively that humans do not have so-called lunate sulci that are homologous with the Affenspalte (Allen et al., 2006). Furthermore (and significantly), “The view that the human lunate sulcus is simply homologous with that seen in other primates has led us to underestimate the extent and importance of occipital reorganization that has occurred in hominid evolution” (Allen et al., 2006, p. 875).

The most parsimonious explanation for why humans do not have lunate sulci is that the Affenspalte was lost during hominin evolution as brains increased in size and in their connectivity between BA 17 and other regions. The Affenspalte may, or may not, have been lost in conjunction with relaxation of surface tensions that previously separated BA 17 from adjacent association cortices, consistent with Van Essen's (1997, 2007) tension-based theory of sulcal patterns. However, this hypothesis may need modification in light of recent research on ferret brains (Xu et al., 2010; Zilles et al., 2013). In particular, the tension-based hypothesis would benefit from exploration of its interaction with another hypothesis (the gray matter hypothesis), which examines cortical folding in light of cellular development of the cortex and its connectivity. Thus, “we propose that the mechanistic influences of fiber tracts and the intrinsic organization and ontogenetic development of the cerebral cortex are related, rather than alternative, processes that enable the progressive differentiation of the cortex during evolution and ontogeny” (Zilles et al., 2013, p. 275). In any event, because the lunate sulcus seems to have been lost during hominin evolution (Allen et al., 2006), it would be appropriate to resurrect the term “Affenspalte” for apes and to drop “lunate sulcus” for humans.

### Repercussions of smith's theory for paleoneurology

Smith chaired Anatomy at the University College London from 1919 to 1937. A charismatic mentor, he placed Joseph Shellshear as Chair of Anatomy at the University of Hong Kong in 1922; Raymond Dart as Chair of Anatomy at the University of Witwatersrand in Johannesburg, South Africa in1923 (where he subsequently named *Australopithecus africanus*); and Davidson Black as Head of Anatomy at Peking Union Medical College in China in 1924 [where he later named *Sinanthropus pekinensis* (now *Homo erectus*)]. All three protégées defended or reiterated Smith's homology of the ape Affenspalte with a human lunate sulcus. Referring to a “voluminous literature… of a controversial nature” about the proposed homology (Black, 1915, pp. 131–132), Black emphasized the importance of the stria Gennari for affirming Smith's hypothesis, but resorted to special pleading to rationalize why this feature was not always bordered by the lunate sulcus in humans. Having examined over 400 Chinese brains, Shellshear also defended Smith's ideas and concluded, “The brain of the Chinese appears to be even more primitive, i.e., more directly comparable with the anthropoid brain, in the occipital region than the Egyptian” (Shellshear, 1926, p. 12).

Dart's description of the ape-sized natural endocast of the famous Taung fossil (Dart, 1925) was inspired by Smith, whom he described as “the master, at whose feet I was privileged to sit” (Dart, 1929, p. 163). Dart misidentified the lambdoid suture reproduced on the endocast as a lunate sulcus (Falk, 2009), which he interpreted as an advanced feature because it was located more caudally than the Affenspalte of apes. Despite the fact that Dart's colleagues, including Smith, were dubious about his claims (Falk, 2009, 2011, 2012), a few contemporary paleoneurologists accepted Dart's mistaken identification of Taung's lunate sulcus and, further, claimed that the back end of the hominin brain evolved before other parts—so-called “mosaic brain evolution” (Barton and Harvey, 2000; Holloway, 2001). Contrary to this view, an unpublished manuscript that Dart completed by 1929 reveals that he thought Taung's entire brain was globally reorganized, and that he recognized his mistake about the lambdoid suture (Falk, 2009, 2011). Unfortunately, for paleopolitical reasons (Falk, 2011), Dart's (1929) manuscript, including a full description of Taung's endocast that included 14 sulci in addition to the two he published in 1925, was never published (but see Falk, 2009, for details and some of Dart's previously unpublished illustrations). Perhaps if Dart's manuscript had been published, the controversy about whether or not Taung and other australopithecines had lunate sulci in a humanlike or apelike position would not have lasted so long.

### The current status of the lunate sulcus in *Australopithecus*

**Figure 2:**
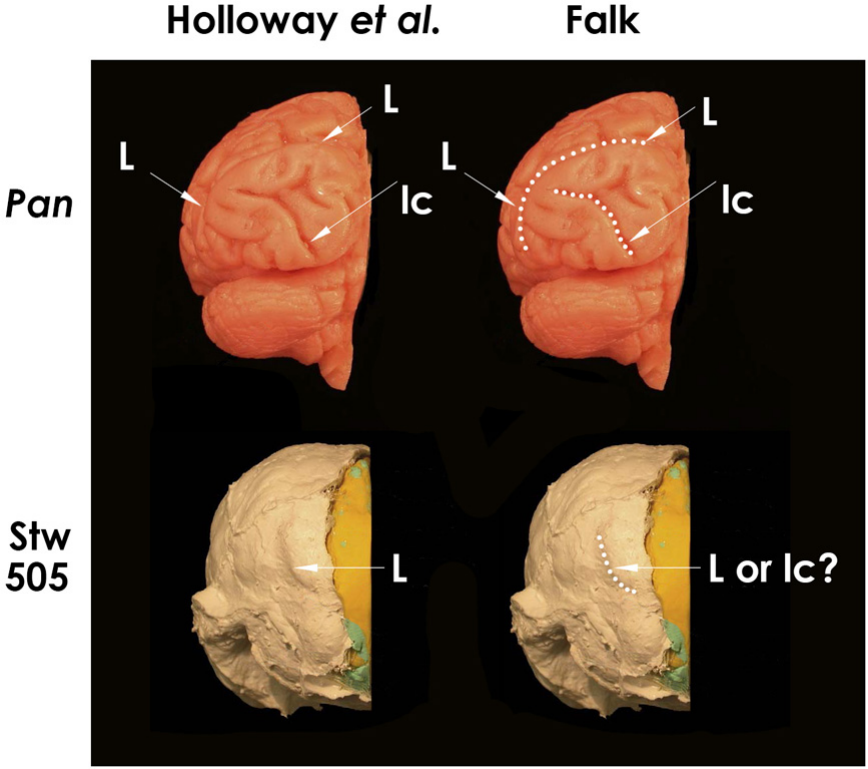
**Posterior views of left hemispheres of a chimpanzee brain cast (above) and the Stw 505 *Australopithecus africanus* partially reconstructed endocast (below), with the midsagittal planes identified by Holloway et al. (2004b: Figure 3) approximately aligned.** Sulci indicated on the left are dotted in for clarity on the right. The identifications on the left are Holloway et al.'s; those on the right are the author's. Abbreviations: *L*, lunate sulcus; *lc*, lateral calcarine sulcus. As indicated on the right, the author confirms Holloway et al.'s sulcal identifications for the chimpanzee brain, but thinks that the sulcus they identified as *L* on Stw 505 looks more like the medial part of *lc* in the chimpanzee brain. Figure after Holloway et al. (2004b), Copyright © 2004, Académie des sciences. Published by Elsevier Masson SAS. All rights reserved.

Holloway et al. stated that “the lambdoid suture on the Taung specimen occludes the possible location of a posteriorly located LS (lunate sulcus), while the typical chimpanzee placement of a lunate on the Taung endocast would violate the parietal sulcal morphology” (Holloway et al., 2004b, p. 290), as first suggested by Clark (1947). In response, Falk showed that space exists on Taung's endocast for a lunate sulcus in an apelike position that would not violate sulcal morphology (Falk, 2009, p. 58 and Figure 6a). Despite these different opinions, most contemporary paleoneurologists now agree that a clear lunate sulcus cannot be identified on the Taung endocast and that “none of the other published australopithecine brain endocasts have a clearly discernible LS” (Holloway et al., 2004b, p. 290). However, an imprint of the lunate sulcus is now claimed to be reproduced in a caudal position on the partially reconstructed Stw 505 endocast from *Australopithecus africanus*, thus confirming Dart's claim about a posterior lunate sulcus in that species (Holloway et al., 2004b). Because Stw 505 is now the *only* australopithecine endocast claimed to support Dart's hypothesis, it deserves scrutiny. Figure 2 (top left) reproduces Holloway et al.'s posterior view of the left hemisphere of a chimpanzee braincast, labeled with their identifications of lunate and lateral calcarine sulci, which I confirm (top right). The lower images reproduce Holloway et al.'s similar view of Stw 505, which their identification of *L* (left), which I question (lower right). I have copies of both casts and, to my eyes, a comparison of Stw 505 with *Pan* (Figure 2, compare bottom and top on the right) shows that the feature Holloway et al. identify as a lunate sulcus in Stw 505 more closely resembles the position and shape of the medial half of the lateral calcarine sulcus of the chimpanzee. (Partial reproduction of sulci is common on hominid endocasts, Clark et al., 1936.) This observation contradicts the authors' statement, “**No other sulcus normally found in the occipital lobe, including the lateral calcarine, inferior and lateral occipital sulci, matches the position or strong posterior crescentic concavity of this sulcus on the Stw505 brain endocast, either in chimpanzee or *Homo***” (Holloway et al., 2004b, p. 4; **emphasis theirs**).

To summarize, Grafton Elliot Smith's assertion that humans have lunate sulci that are homologous with the Affenspalte of apes was incorrect (Allen et al., 2006), as was Raymond Dart's 1925 observation that such a sulcus was visible on the Taung endocast (Holloway et al., 2004b; Falk, 2009, 2011). Had they been right, there would be good reason to believe the Affenspalte migrated caudally during hominin brain evolution and that this trend began by the time of australopithecines (~2.5 to 3.0 mya), despite their small ape-sized brains. It would also be reasonable to accept the lunate sulcus as a potential indicator of cortical reorganization across the entire hominin endocast record (Allen et al., 2006; de Sousa et al., 2010; Gómez-Robles et al., 2013). This seems dubious, however, not only because Smith's and Dart's assertions about the Affenspalte/lunate sulcus turned out to be incorrect, but also because the hypothesis of the migrating lunate sulcus now rests *entirely* on one questionable sulcal identification for one australopithecine endocast (Figure 2). Although it is clear from comparative evidence that the lunate sulcus disappeared as parietotemporo-occipital association cortices evolved and enlarged (Allen et al., 2006), sulcal patterns on endocasts have yet to shed light on when, and in which hominins, this happened.

## Broca's area

**Figure 3:**
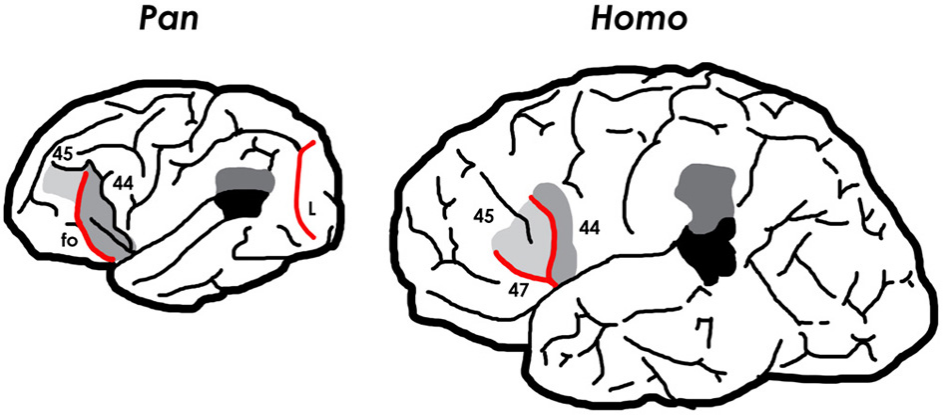
**Left hemispheres of a chimpanzee and human brain illustrating different sulcal patterns.** The chimpanzee has a lunate sulcus (*L*) at the rostral border of the primary visual cortex, which was lost during human evolution. The chimpanzee also has a fronto-orbital sulcus (*fo*) that delimits so-called “Broca's cap” (BA 44 and sometimes part of BA 45). Most of *fo* became buried deep within the brain as hominin brains enlarged. Consequently, the bulge on human brains that appears to occupy the same location as the orbitolateral swelling in chimpanzee frontal lobes, contains BA 47 and BA 45, in addition to two new sulci (the horizontal and ascending limbs of the anterior Sylvian fissure) that form two sides of the pars triangularis (BA 45). The *fo*, *L*, and sulci bordering BA 45 are exaggerated and reddened here.

With few exceptions, the basic sulcal patterns of apes and humans are very similar (Connolly, 1950). As we have seen, the loss of the lunate sulcus is one of the few departures of human brains from the primary sulcal patterns of apes. A second difference is that humans evolved a distinct sulcal pattern in the inferior “third” convolution of both frontal lobes. In the left hemisphere, this pattern is associated with Broca's speech area (Broca's area) which facilitates language production in addition to other activities (reviewed in Schenker et al., 2010) (Figure 3). Broca's area and its homolog on the right hemisphere consist cytoarchitecturally of BA 44 (pars opercularis) and BA 45 (pars triangularis). Although three-dimensional volumetric studies of these two regions show that sulci are not reliable landmarks of their cytoarchitectonic borders (Amunts et al., 1999), paleoneurologists are necessarily confined to interpreting only the free surface of these (and other) regions on endocasts. Fortunately, on the surface of human brains there “are regions, i.e., the free surfaces of the triangular and opercular parts, in which the probability is very high of localizing areas 45 and 44, respectively” (Amunts et al., 1999, p. 339). The free surface of the pars triangularis is, thus, bordered by the pylogenetically new horizontal and ascending branches of the Sylvian fissure, while the surface of the pars operularis is located caudal to the latter branch (Figure 3).

Although chimpanzee frontal lobes contain cytoarchitectonic homologs of BA 44 and BA 45 (Sherwood et al., 2003; Schenker et al., 2010), these are associated with a sulcal pattern that differs completely from the derived pattern of humans (Figure 3). Chimpanzee brains (and, indeed, those of all great apes) have a fronto-orbital sulcus (*fo*), not seen in human brains, which forms the anterior boundary of a bulge that, historically (Bailey et al., 1950; Connolly, 1950), has been recognized as fronto-parietal operculum (i.e., BA 44), in contrast to the surface of human brains in which an ascending ramus of the Sylvian fissure borders BA 44 rostrally and BA 45 caudally (Amunts et al., 1999) (Figure 3). Recent cytoarchitectonic studies, however, suggest that the precise relationship between sulci and the boundaries between BA 44 and BA 45 is more variable on the surface of chimpanzee brains (Sherwood et al., 2003; Schenker et al., 2010) than on the surface of human brains (Amunts et al., 1999). Nevertheless, a recent analysis of the relationship between BA 44, BA 45, and sulci on the surface of both hemispheres from the brains of 12 chimpanzees found that BA 45 was most often located anterior to *fo* and sometimes above BA 44, while BA 44 was most typically located anterior to the precentral inferior sulcus (*pci*) (Schenker et al., 2010) (see Figure 3), although “there was extensive interindividual variation in the precise boundaries of these cortical areas relative to the position of sulcal features” (Schenker et al., 2010, p. 735).

Despite the growing consensus that the sulci of the inferior frontal gyrus are not reliable indicators of the *precise* divisions between BA 44 and BA 45 for chimpanzees (Sherwood et al., 2003; Keller et al., 2009; Schenker et al., 2010), it is informative to compare the external morphology of chimpanzee and human frontal lobes in this general region because of their distinctive sulcal patterns. As noted, in humans derived horizontal and anterior branches of the Sylvian fissure enclose a frontal operculum (pars triangularis) that is not seen in ape brains, although they have a BA 45, which is not an operculum (i.e., does not cover the insula). The only operculum apes have is the frontal-parietal operculum, which is caudal to *fo* which is now known to consist of all or part of BA 44 and sometimes part of BA 45 (Schenker et al., 2010). Based on comparative and embryological evidence, Connolly hypothesized that, as frontal association cortices expanded during hominin evolution, *fo*, at least in its lower part, became the anterior limiting sulcus of the insula deep within the brain, which accounts for the absence of this sulcus on the cortical surface of human brains (Connolly, 1950, p. 330). Connolly further suggested that the human pars triangularis evolved from a wedge-shaped mass of cortex (presumably BA 45) that expanded downward and backward, between the developing orbital operculum (BA 47) and fronto-parietal operculum (BA 44), from which it was separated by two new sulci – namely, the horizontal and ascending rami of the Sylvian, respectively (1950, pp. 159, 330). Although this hypothesis seems reasonable (see Figure 3), it has yet to be addressed by contemporary comparative neuroanatomical and embryological researchers. For their part, paleoneurologists would like to determine where in the fossil record of hominin endocasts, a humanlike sulcal pattern emerged on the surface of the inferior frontal convolution. Unfortunately, the small sulci that delimit two sides of BA 45 on the cortical surface of human brains do not reproduce well on endocasts. *Fo*, on the other hand, reproduces well on ape brains and, often, on their endocasts.

Human and chimpanzee brains both have an orbital bulge near the caudal end of the frontal lobe at the level of the temporal pole, which is known as the cap, orbital cap, or Broca's cap. As Connolly documented long ago (1950, p. 326), comparative cytoarchitecture of these caps in apes and humans shows that they are not homologous. The cap of humans contains BA 47 (pars orbitalis) and, above that, BA 45 (pars triangularis), but not BA 44 which shifted caudally relative to the temporal pole during hominin evolution. Although the cap of chimpanzees appears in the same general location, it typically contains BA 44 and sometimes part of BA 45 (Sherwood et al., 2003; Schenker et al., 2010) (Figure 3). For this reason, the practice of homologizing the caudal orbitolateral bulges of the frontal lobes of apes, fossil hominins, and extant humans as so-called “Broca's cap” or, more simply, the “orbital cap” is questionable. This observation is consistent with a recent histological study of BA 44 and BA 45 in chimpanzees, which raises questions about assertions of humanlike leftward asymmetry in the surface area of the inferior frontal convolution in great apes (Schenker et al., 2010; see also Keller et al., 2009). (See Holloway et al., 2004a, pp. 31–32 for discussion of asymmetries of Broca's cap in humans.) Despite these findings and the fact that the small sulci that border the pars triangularis are not, as a rule, reproduced on hominin endocasts, one hopes that future comparative studies in conjunction with analyses of the relevant regions on small to medium-sized endocasts from early *Homo* will eventually shed light on the transition from apelike to humanlike sulcal patterns associated with the evolution of Broca's speech area.

## Prefrontal cortex

Analyses of sulcal patterns on the dorsolateral surface of the frontal lobes of great apes, humans, and australopithecines are more promising than the paleoneurological studies of sulci in the occipital lobe and inferior frontal convolution described above, especially when interpreted in light of recent findings about the connectivity of the prefrontal cortices in humans and apes (Semendeferi et al., 2011). The middle frontal sulcus (*fm*) deserves particular attention because it occurs in brains of living great apes and humans in primitive and derived forms, respectively (Connolly, 1950) and because, unlike the sulci discussed above, it happens to reproduce well on australopithecine endocasts.

**Figure 4:**
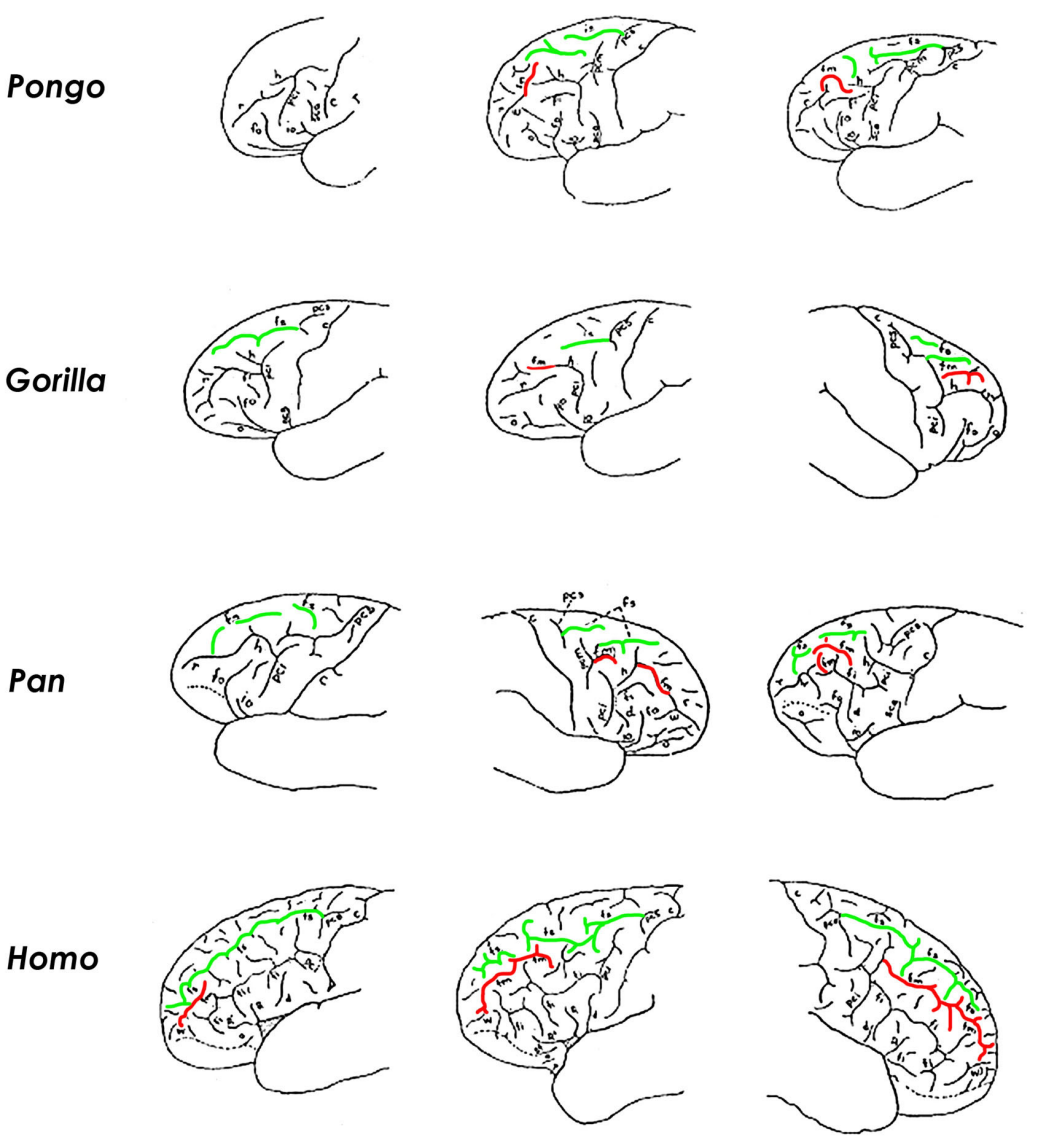
**The superior and middle frontal sulci in brains of great apes and humans.** For each genus, illustrations are arranged from the simplest (left) to the most complicated (right) manifestation of *fm* (reddened). In the ~20% of great ape hemispheres that have a separate *fm*, it is short and irregular in shape (right column). Human brains, on the other hand, usually have a separate elongated *fm* that courses parallel and lateral to the superior frontal sulcus (*fs*, in green) (bottom row). According to Connolly, the caudal end of human *fm* emerged in conjunction with evolutionary expansion of prefrontal association cortex (i.e., it is a phylogenetically new portion of *fm*). These illustrations are modified from Connolly (1950). Those for *Pongo* are, left to right, from pages 78, 71, and 71; *Gorilla*, pages 99, 99, and 91; *Pan*, pages 113, 108, and 114; *Homo*, pages 188, 189, and 202. Identifications of sulci and their abbreviations from Connolly (1950): *c*, central; *d*, diagonal; *fi*, inferior frontal; *fm*, middle frontal; *fo*, fronto-orbital; *fs*, superior frontal; *h*, horizontal branch of *pci*; *io*, opercular; *o*, orbital; *pci*, inferior precentral; *pcm*, middle precentral; *pcs*, superior precentral; *r*, rectus; *R*, ascending branch of Sylvian; *R'*, horizontal branch of Sylvian; *sca*, subcentral anterior; *sf*, subfrontal; *W*, fronto-marginal of Wernicke.

*Fm* appears in some brains representing all of the great apes (Figure 4). In those ape brains that have *fm*, it is homologous with the caudal end of the rectus sulcus (*r*) of monkeys (Eberstaller, 1890; Connolly, 1950). The form of *fm* varies in its complexity within each ape genus, as discussed and illustrated by Connolly (1950) (Figure 4). In great apes that have an *fm*, it may be attached to *r*, form one or (rarely) more separate dimples or small sulci, and/or be attached to the horizontal branch (*h*) of the precentral inferior sulcus (*pci*, which is the homologue of the horizontal branch of the arcuate sulcus of monkeys) (Connolly, 1950). Of the 24 great ape hemispheres illustrated by Connolly, only five (21%) had a separate *fm*, and these were fragmentary or very small. Figure 4 illustrates variations of *fm* illustrated by Connolly for each of the great apes and humans, and includes examples of the most simple to most differentiated forms (left to right) for each genus.

Unlike apes, a separate *fm* in human brains is “a constant and important sulcus present in all specimens” (Connolly, 1950, p. 203). Although its rostral part is homologous with the posterior (sagittal) part of *r* of apes, Connolly recognized the greater (caudal) portion of the human *fm* as a new sulcus that emerged with evolutionary expansion of prefrontal association cortices (Connolly, 1950). Connolly noted that the phylogenetic emergence of *fm* as a consistent and elongated sulcus in humans was accompanied, not only by frontal lobe expansion, but also by a change in shape in which, compared to apes, “the orbital margin has grown downward and outward increasing anteriorly the breadth of the lobe” (Connolly, 1950, p. 75).

Connolly also detailed gradations of complexity of *fm* that correlated with development of the frontal lobe as a whole in the 120 human hemispheres that he studied (Figure 4). In contrast to great apes, the human *fm* courses in a sagittal direction lateral and approximately parallel to the superior frontal sulcus (Figure 4). As it courses caudally, the human *fm* runs through the center of the middle frontal gyrus where it is surrounded by dorsolateral prefrontal association cortices including Brodmann's areas 10, 46, 9, and 8. Evolutionary enlargement of the frontal cortex in apes and humans has involved mostly dorsolateral prefrontal cortex (Van Essen, 2007), which is associated with “executive functions,” including organizing input from various senses, maintaining attention, monitoring working memory, and coordinating goal-directed behaviors. “Together, these abilities would have been necessary for navigating both the complex social groups and unpredictable, dangerous environments of our hominin ancestors. Thus, the capacities enabled by the PFC, while most are not exclusively human, are certainly a crucial aspect of what we think of as “human cognition”” (Teffer and Semendeferi, 2012, p. 192). This observation is consistent with cytoarchitectural evidence from apes and humans, which suggests that, compared to other parts of the brain that the authors studied, the evolution of hominin prefrontal cortex was characterized by a differential increase in the width of its minicolumns and complexity of its interconnectivity between neurons (Semendeferi et al., 2011).

### The middle frontal sulcus of *Australopithecus*

**Figure 5:**
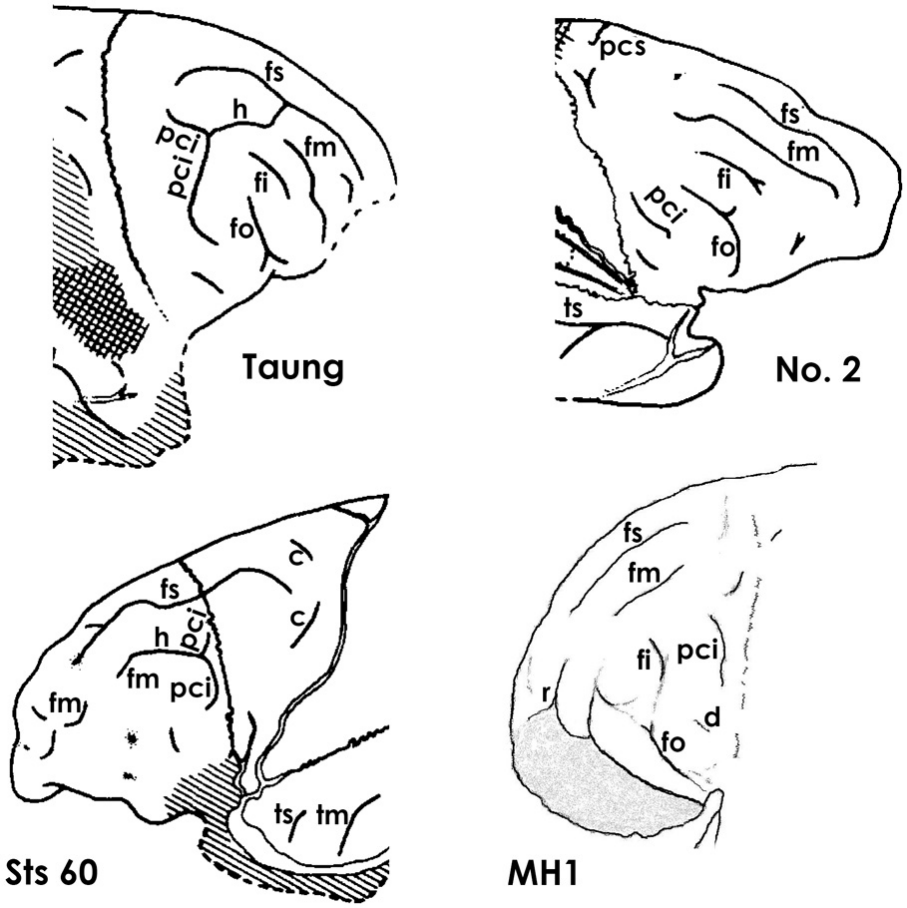
**The superior and middle frontal sulcus in *Australopithecus* endocasts.** The middle frontal sulcus (*fm*) of MH1 (*Australopithecus sediba*) is identified here because (1) of its relationship to *r* (not identified by Carlson et al., 2011), (2) it does not appear to have been derived from (or proximal to) *pci* (which *fi* always is, Connolly, 1950), and (3) it courses approximately in the middle of the prefrontal cortex (hence its name). Taung, No. 2, and Sts 60 are endocasts from *Australopithecus africanus*. Note that all four australopithecines have separate branches of *fm*, which are rare in ape brains but typical of human brains, and that, in all four, *fm* is lateral to a long superior frontal sulcus (*fs*). Identifications of sulci: *tm*, middle temporal; *ts*, superior temporal; see legend to Figure 4 for other sulcal abbreviations. All illustrations except for MH1 are reproduced from Falk (1980). The identification of *fi* in Taung and No. 2 departs from Falk's (1980, 2009) earlier identification of that sulcus as *r*, but agrees with those of Dart (1929) and Schepers (1946). The line drawing for MH1 is based on the unlabeled photograph of the MH1 endocast from Carlson et al. (2011), reproduced on the left side of Figure 6. Compare the identifications for MH1 provided here with those of Carlson et al. reproduced on the right side of Figure 6.

**Figure 6:**
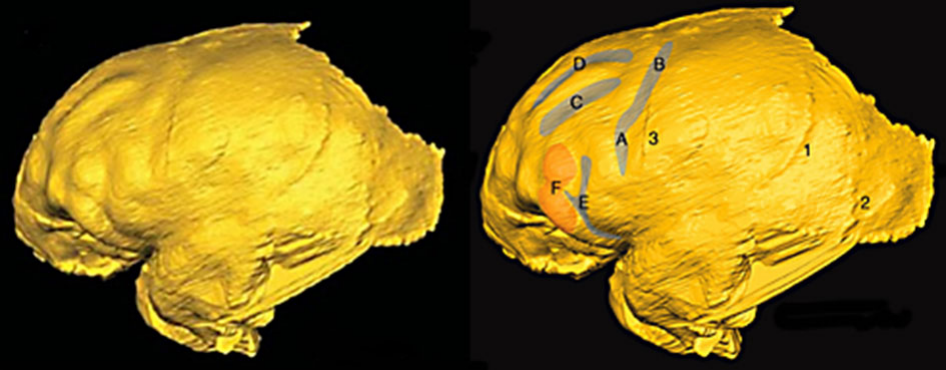
**Left lateral view of virtual endocast of MH1.** Image on right shows Carlson et al.'s identifications: (A) *pci*, (B) *pcs*, (C) *fi*, (D) *fs*, (E) *fo*, (F) anterior inferior frontal gyrus, (1) and (2) meningeal arteries, (3) coronal suture. See legend to Figure 4 for key to abbreviations of sulci. Images reproduced from Carlson et al. (2011). Reprinted with permission from AAAS.

Fortunately, the fossil record of australopithecine endocasts is clearer for *fm* than it is for sulci in the inferior frontal convolution of the frontal lobe or the lunate sulcus. Unlike most ape brains, a separate *fm* occurs in the four *Australopithecus* endocasts for which relevant sulcal patterns have been published (Figure 5), and it courses lateral and approximately parallel to the superior frontal sulcus on the frontal lobes in three of them (details are reproduced less clearly on the Sts 60 endocast). These include three *A. africanus* endocasts: Taung (Dart, 1929; Falk, 1980, 2009; Holloway et al., 2004a), Sts 60 (also called the No. 1 endocast from Sterkfontein; Schepers, 1946; Falk, 1980; Holloway et al., 2004a), and the No. 2 endocast from Sterkfrontein (Schepers, 1946; Falk, 1980; Holloway et al., 2004a). A fourth australopithecine endocast, MH1 from *A. sediba*, also reproduces this pattern, although *fm* was identified as the inferior frontal sulcus (*fi*) in the original description of the endocast (Carlson et al., 2011) (Figure 6). My copy of the MH1 endocast suggests that the sulcus identified as *fm* in Figure 5 cannot be *fi*, however, because MH1 has an obvious rectus sulcus (*r*) (not identified in the original description) that arcs from in front of the sulcus down toward the frontal pole (the typical position of *r* relative to *fm*, but not *fi* Connolly, 1950). The sulcus in question (C in Figure 6) also does not appear to be derived from (or proximal to) the lower part of *pci* (A in Figure 6), as is always the case for *fi* of apes that have the sulcus and humans (Connolly, 1950), but instead seems to course higher in a caudomedial direction toward *pcs* (B is Figure 6) rather than in a more horizontal direction toward *pci* (A), as is typical for *fi* of apes and humans (Connolly, 1950). As Connolly discussed and illustrated in light of comparative sulcal patterns, and with reference to cytoarchitecture (1950, pp. 106–108), *fi* may or may not be connected directly with the lower part of *pci* in ape and human brains, but its caudal end is always proximal to *pci* and it courses in the inferior rather than the middle part of the lateral surface of the frontal lobe (Connolly, 1950, pp. 106–108, 193–194). The fact that the sulcus Carlson et al. identify as *fi* is located approximately in the middle of the frontal lobe (which is why *fm* is named the middle frontal sulcus), as well as the fact that it courses lateral and parallel to *fs* (D in Figure 6) is further evidence that it is, indeed, *fm*. In sum, *fi* may or may not be present in ape brains (Connolly, 1950, p. 110), but, when it is, it is located at a level that courses directly above the orbitofrontal sulcus (*fo*) (i.e., not as far away as C is from E in Figure 6. For these reasons, I believe MH1's prefrontal cortex has a derived *fm* similar to those of the other australopithecines (Figure 5). Indeed, if it lacked *fm*, the frontal lobe of *Australopithecus sediba* would be less derived toward a human condition than those of the other australopithecines.

Carlson et al. identified *fm* as *fi* in MH1 because of “its position, orientation, and close association with the superior portion of *the fronto-orbital sulcus*,” and claimed that this identification was in keeping with “interpretations of similarly positioned sulci on chimpanzee brains” by Clark et al. (1936); Walker and Fulton (1936); Bailey et al. (1950); Rilling et al. (2008), and Schenker et al. (2010) (Carlson et al., 2011, p. 1404). The following information is in response to an anonymous reviewer's request that this claim be addressed: Some of the references cited by Carlson et al. are very old and used terminology that is now antiquated, as detailed by Connolly (1950). Clark et al. (1936), for example, did not recognize the existence of *fm*, so did not label the *fm* that appear in their illustrations of 11 hemispheres from six chimpanzee brains. For some specimens, their identification of *fi* is correct (e.g., right hemisphere of chimpanzee 3, in which an obvious unlabeled *fm* appears above *fi*); in others the authors have mistakenly identified an obvious *fm* as *fi* (left hemisphere of chimpanzee 3). Walker and Fulton (1936) used similar outdated terminology (Connolly, 1950, pp. 116–117) that did not recognize *fm*. Nevertheless, they correctly identified *fi* (their IF) on photographs from four hemispheres of three chimpanzees (Pau, Josephine, and Bonzo). All of the *fi* appear to have stemmed from, or be proximal to, the lower part of *pci* (Walker and Fulton's PRI) rather than coursing caudally and upward toward *pcs* (Walker and Fulton's PS), contrary to Carlson et al.'s identification of *fi* for MH1 (Figure 6). Further, Walker and Fulton's photographs for the right hemispheres of Pau and Josephine show clear but unlabeled *fm* above *fi*. Contrary to Carlson et al., Bailey et al.'s identifications and descriptions of both *fi* and *fm* in chimpanzees (1950: Figure 4a) are consistent with Connolly (1950) as well as the identifications provided here for MH1 (Figure 5). As noted (and illustrated) by Bailey et al., “the inferior frontal sulcus, when clearly developed, runs from the inferior precentral sulcus” (1950, p. 29), which is not the case for the sulcus that Carlson et al. identify as *fi* (Figure 6). Nor do the more modern references cited by the authors support their identification. The schematic that illustrates frontal lobe sulci of *Pan* in Schenker et al. (2010: Figure 1) includes both *fi* and *fm* and is consistent with the identifications of those sulci presented here for MH1. The schematic in Rilling et al. (2008: Figure 2b), on the other hand, does not include *fm*. Although it is difficult to assess the sulci, which are imposed on a schematic summary of three-dimension tractography results from several chimpanzees, the *fi* identified by Rilling et al. appears to be proximal to *pci* and extends further forward than does the *fi* identified for MH1 by Carlson et al. In sum, the literature cited by Carlson et al. is consistent with an identification of *fm* rather than *fi* for the sulcus labeled C in Figure 6. In my opinion, this is also true for the surface renderings from *in vivo* MRI scans of eight adult chimpanzees shown in Carlson et al.'s Supporting Online Material (Figure S2), on which the authors labeled *fi* but not *fm*, although *fm* is clearly visible on some of them in a position comparable to that of C in Figure 6.

### The inferior frontal gyrus of Australopithecus

**Figure 7:**
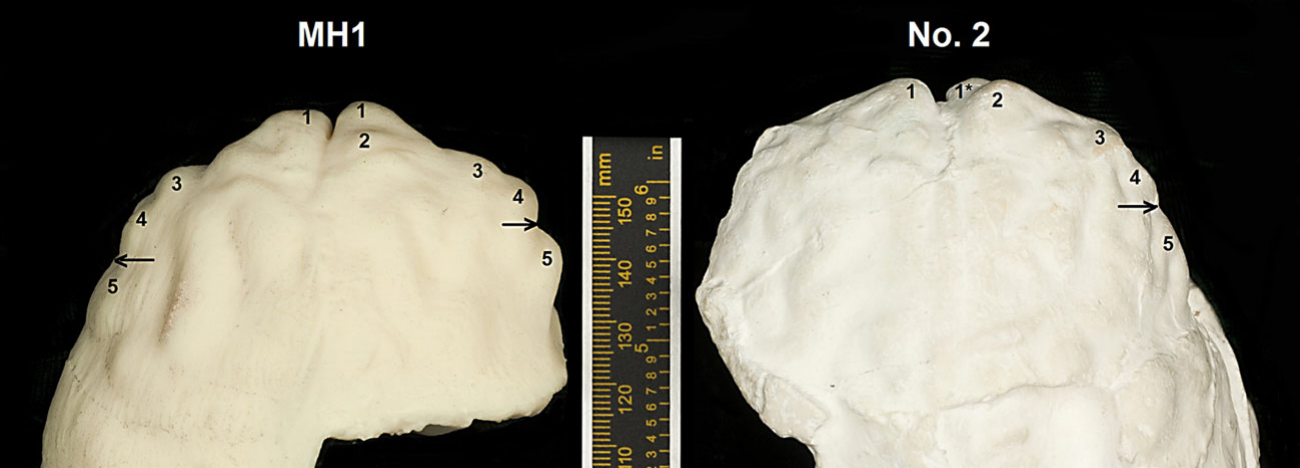
**Dorsal view of frontal lobes of MH1 (*Australopithecus sediba*) and the No. 2 endocast from Sterkfontein (*Australopithecus africanus*).** Numbers indicate comparable shape features: 1, frontal poles; 1^*^, fragment of frontal pole; 2, swelling above the right frontal pole near the superior sagittal sinus; 3,4, double bumps on perimeter of frontal lobe that are relatively lateral to the frontal poles, giving the rostral ends of the endocasts a squared-off appearance (i.e., they are wider rostrally) compared to *Paranthropus* and chimpanzees; 5, bulge above and caudal to the level at which the fronto-orbital sulcus incises the orbitolateral border of the frontal lobe (indicated by arrows). The endocast of MH1 is a hard print of a virtual endocast; that of No. 2 is a plaster copy of a natural endocast.

Carlson et al. correctly noted that the frontal lobe convolutions of MH1 are generally apelike and appear comparable to those of the No. 2 specimen and Sts 60, to which I would add Taung (Figure 5). The authors shaded part of the anterior inferior frontal gyrus (F in Figure 6) and observed that “the shape of the MH1 inferior frontal gyrus clearly differs anteriorly from the ape condition and also from other South African australopith endocasts, except perhaps for Sterkfrontein Type 2, which does not preserve the comparable area” (2011, p. 1405). However, the other australopithecine endocasts do not preserve the orbital surface of the frontal lobes, so cannot be compared to MH1 in this region. Further, when one compares the right sides of the No. 2 endocast and MH1, the latter has the same sulcal pattern and general shape as the former (Figure 7). All of the australopithecine endocasts that reproduce sulci in the frontal lobe have similar sulcal patterns, including fragments of sulci above and proximal to *fo* that appear to be elements of *fi* (Figure 5). For these reasons, the endocast of MH1 appears to be similar to those of other australopithecine endocasts that reproduce comparable details in comparable regions.

Significantly, Carlson et al. suggested that F in Figure 6 might represent early stages in interconnectivity in BA 45 that preceded the emergence of a human-like pars triangularis, which is an intriguing hypothesis that may pertain to all australopith endocasts. The comparative cytoarchitectonic studies discussed above suggest that the lower part of F may be BA 47 while the superior part might represent BA 45. If so, expansion of australopithecine endocasts in this region (which is manifested in a squared-off shape when viewed dorsally, see below) could indicate reorganization associated with an eventual insertion of a wedge leading to the pars triangularis in humans (discussed above). Consistent with this, Connolly suggests that the upper part of *fo* may be analogous (not homologous) to the horizontal branch of the Sylvian fissure, which forms the anterior boundary of the pars triangularis in humans (Connolly, 1950, p. 330). Carlson et al.'s hypothesis, which should be considered generally for *Australopithecus* endocasts (see below), is an important one that deserves investigation with future comparative cytoarchitectonic studies in apes and humans of BA 45, BA 44 and, in particular, BA 47.

Other paleoneurological evidence supports the hypothesis that prefrontal cortex began to reorganize at some unknown point prior to the ~2.5 to 3.0 million-year-old dates for *Australopithecus africanus* (*Australopithecus sediba* is dated at ~1.9 my). Consistent with Connolly's observations about shape changes that accompanied the derivation of the caudal portion of *fm* during hominin evolution, and consistent with Carlson et al.'s hypothesis that the gross morphology of the australopithecine inferolateral frontal lobe may represent a transitional stage toward a humanlike frontal operculum, the orbital margin of *Australopithecus* is expanded downward and outward, which increased the breadth of the prefrontal cortex, giving its rostral perimeter a relatively squared-off shape in dorsal view compared to the more distant early human relative, *Paranthropus* (Falk et al., 2000). These findings, together with those from comparative cytoarchitecture (Schenker et al., 2010; Semendeferi et al., 2011), raise the fascinating question of how long ago (and why) the hominin prefrontal cortex first began to reorganize. Hopefully, future discoveries of hominin endocasts that predate *Australopithecus africanus* will shed light on this matter.

## Summary and conclusions

During human brain evolution, association cortices expanded and reorganized caudally in the parietotemporo-occipital region and rostrally in (at least) the middle and inferior frontal gyri of the frontal lobes. This paper addresses what can, and cannot, be gleaned about the evolution of association cortices from hominin endocasts combined with comparative cytoarchitectonic evidence from extant apes and humans. Starting at the back of the brain, we reviewed the historical debate about whether or not the so-called lunate sulcus is a reliable landmark for determining the lateral representation of primary visual cortex (as an indicator of relative expansion of nearby association cortices) on human brains and hominin endocasts. Since humans do not have sulci that are homologous with the Affenspalte of apes (Allen et al., 2006), contrary to an hypothesis that has dogged paleoneurology for over a 100 years (Smith, 1903), it would be best to drop the label lunate sulcus in reference to human brains and to resurrect the term Affenspalte for apes. Although the general claim that australopithecine endocasts reproduce lunate sulci in posterior (supposedly) humanlike locations is no longer accepted, this assertion continues to be made for one australopithecine endocast (Stw 505). However, as shown above, there is reason to question the interpretation of a lunate sulcus in this specimen (Figure 2). In any event, interpretations of the back end of hominin endocasts have contributed to an unfortunate, prolonged controversy and, so far, have not been particularly constructive for investigating cortical reorganization in hominins.

We have better luck moving forward in the brain to a region of the frontal lobe that, in humans, constitutes Broca's speech area in left hemispheres. As discussed and illustrated above, humans have a derived sulcal pattern in this region that differs from that shared by great apes and australopithecines. Exciting recent comparative cytoarchitectonic studies have implications for interpreting both the sulcal and gross morphology of the inferior frontal gyrus on hominin endocasts. Nevertheless, one should refrain from homologizing the orbitolateral bulge that is located approximately at the level of the temporal poles in ape and human brains as so-called Broca's cap because the cytoarchitecture in this region differs in the two groups. New information shows that *Australopithecus* endocasts reproduce a middle frontal sulcus that is derived toward a humanlike condition compared to apes, which suggests expansion and reorganization had begun in the middle frontal gyrus by at least the time of *Australopithecus africanus* (i.e., ~2.5 to 3.0 mya). The shape of the inferior frontal gyrus in australopithecines is also derived in ways that are consistent with a similar trend for that part of the brain, perhaps in conjunction with reorganization of BA 45 (Carlson et al., 2011), BA 47, and BA 44. This paper also presents new information about the frontal lobe of MH1 (*A. sediba*), and concludes that it is similar to the frontal lobes of other australopithecines. The discussion of australopithecine endocasts provides an example of how the few sulci that are reproduced clearly on hominin endocasts may be interpreted within a comparative framework that incorporates details about sulcal patterns, overall brain shape, and cytoarchitecture in apes and humans. With luck, such an approach well advance the field of paleoneurology, and may help counter the misperception that those who study endocasts from early human relatives are engaging in paleophrenology.

### Conflict of interest statement

The author declares that the research was conducted in the absence of any commercial or financial relationships that could be construed as a potential conflict of interest.
